# Polypropylene Blends for Highly Drawn Tapes with Improved Toughness

**DOI:** 10.1021/acsomega.3c01772

**Published:** 2023-06-15

**Authors:** László József Varga, Ákos Görbe, Tamás Bárány

**Affiliations:** † Department of Polymer Engineering, Faculty of Mechanical Engineering, Budapest University of Technology and Economics, Műegyetem rkp. 3., H-1111 Budapest, Hungary; ‡ MTA-BME Lendület Lightweight Polymer Composites Research Group, Műegyetem rkp. 3., H-1111 Budapest, Hungary

## Abstract

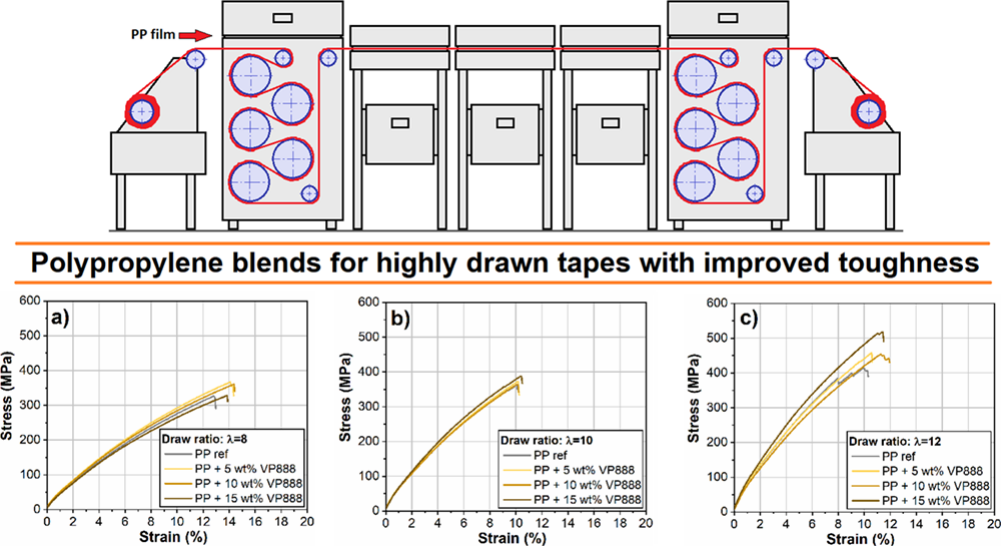

In this study, we used four amorphous poly-alpha-olefin (APAO) grades to improve the toughness of drawn polypropylene (PP) tapes. The samples containing different amounts of APAOs were drawn in a heat chamber of a tensile testing machine. The APAOs reduced the work of drawing and increased the melting enthalpy of the drawn specimens, as they facilitated the movement of the PP molecules. The APAO with the highest molecular weight and with a low level of crystallinity increased both the tensile strength and the strain-at-break of the specimens, so we also produced drawn tapes from that PP/APAO blend on a continuous-operation stretching line. The continuously drawn tapes also showed improved toughness.

## 1 Introduction

Polypropylene (PP) is one of the most widely used commodity plastics nowadays. Its versatility is ensured by its low price, easy processability, low density, and excellent chemical resistance. PP, however, has only modest mechanical properties. This problem can be overcome with reinforcement. The most common reinforcements are short glass fibers, which makes it possible to use PP for industrial applications.^1^ Another possibility for increasing the properties (most importantly, tensile strength and modulus) is to exploit the polymer’s tendency to molecular orientation.^2−5^

Molecular orientation is a unique state of the polymer materials when the chain molecules reside more-or-less parallel to each other, which dramatically—often by two orders of magnitude—increases the polymer’s strength in the direction of the orientation.^6−8^ Such orientation can be achieved in different processes, such as gel-spinning, melt-spinning, and solid-state drawing. During gel-spinning, orientation is achieved in a polymer solution, and it is usually applied for polymers with very high molecular weight, as such polymers that cannot be melted due to the immense amount of secondary bonds between their huge molecules.^9^ Thus, gel-spinning is mostly used to produce high-end fibers with exceptional strength and modulus,^9^ but it also gained importance in producing nanofibers.^10^ During melt-spinning, fibers are formed in the melt state. Melt-spinning has been used to produce synthetic fibers for the textile industry using a wide range of polymers.^9^

In the case of solid-state drawing, a preexisting polymer structure—fiber or tape—is drawn at an elevated temperature. In industrial processing, the tapes to be drawn are usually produced with sheet film extrusion or film blowing, with the former being the predominant method; as in the case of sheet film extrusion, the initial parameters of the film can be controlled more precisely with different cooling methods (e.g., cooling cylinders, water bath). Biaxially oriented films, however, can only be produced by film blowing. An elevated temperature is needed during the drawing process, which should not exceed the melting temperature of the particular polymer grade, to provide enough mobility to the molecules. The most important quantity in solid-state drawing is the draw ratio (λ), i.e., the ratio of the length of drawn and undrawn fibers or tapes. In the case of PP tapes, usually a draw ratio between λ = 12 and λ = 15 is applied in industrial production to achieve the most beneficial properties.^11−13^ During the drawing process, the internal stresses caused by the drawing forces drive the polymer molecules to move parallel next to each other. In semicrystalline polymers, however, the crystalline and the amorphous phases behave differently during drawing. The crystalline phase, which originally forms spherulites or lamellae, goes through a complex process called micronecking, first described by Peterlin,^14^ and forms long fibrils.^14−16^ The molecules of the amorphous phase, which originally did not show any defined arrangement whatsoever, are settled in a more-or-less parallel manner due to the increased molecular mobility.^17^ There are also tie molecules, however, that go through both the amorphous and the crystalline phases. As these molecules are also part of the crystalline phases, they are constrained, and they become taut tie molecules during drawing.^6,14−18^

In this model, the orientation of the amorphous and the crystalline phases can be differentiated. Yamada et al.^19^ showed that in the case of drawn PP tapes, crystalline orientation reaches its peak at around the draw ratio of λ = 9, and at higher draw ratios, only the orientation of the amorphous phase increases.^19^ Since then, several studies have showed the orientation of the crystalline phase is finished around the draw ratio of λ = 9^17,20^ The degree of orientation is usually measured with wide angle X-ray diffraction. This method, however, only provides the orientation of the crystalline phase, but combined with other methods (e.g., sonic velocity measurement or optical birefringence measurement), the orientation of the amorphous phase also can be obtained. The degree of orientation is usually described by the Hermans orientation function, which is a dimensionless number, and it can vary between −0.5 and 1. If the orientation function is 1, that means a theoretically perfect orientation (i.e., all the molecular segments are parallel to the direction of the measurement), while the value of −0.5 means that they are all perpendicular to it. In the case of completely random orientation, the orientation function is 0. The amorphous orientation increases the tensile modulus much more than the tensile strength; thus, above the draw ratio of approximately λ = 9, the maximum strain of the drawn tapes is reduced while their strength is only moderately increased.^20^ This behavior can hinder the application of drawn PP tapes in many industrial segments where toughness is also important besides the increased strength of the drawn tapes.^20,21^ This problem could be overcome with the use of blends of PP and amorphous poly-alpha-olefins (APAOs) to produce drawn tapes.^22^

Amorphous APAOs are similar to atactic PP (aPP), as they mostly consist of propylene repeating units residing in an atactic manner, but, contrary to the aPP, APAOs are not a byproduct of isotactic PP (iPP) production; they are produced on purpose to be used as melt adhesives or sealants. This makes it possible for the producers to tailor-make their properties, i.e., molecular weight, viscosity, melting temperature, etc. In the APAOs, propylene is sometimes copolymerized with larger alpha-olefins (e.g., hexene or octane), and they usually contain plasticizers (e.g., wax).^23^ APAOs have an unlimited solubility in PP, however, due to their atactic nature, their molecules can only reside in the amorphous phase of the PP.^24^ Chen et al.^24,25^ investigated the properties of iPP/aPP blends, and they found that a small amount of aPP increased the crystallinity of the blend, but the overall crystallinity decreased with increasing aPP content. The normalized crystallinity of the PP phase, however, increased with increasing aPP content. They concluded that this phenomenon was caused because the tiny molecules of aPP acted like a diluent, and they increased the mobility of iPP molecules and reduced the entanglements between them.^24,25^ In this study, our goal is to exploit this property of APAOs—which are very similar to aPP—to increase the maximal strain of drawn PP tapes without the cost of reducing tensile strength.

## 2 Experimental Section

We used the Tipplen H681F (MOL Petrolkémia Zrt., Tiszaújváros, Hungary) highly isotactic PP homopolymer as the matrix material, with a melt flow index of 1.7 g/10 min (measured at 230 °C with 2.16 kg). The APAOs used were propene-rich melt adhesives, and they are marketed under the tradename of Vestoplast 708, Vestoplast 750, Vestoplast 792, Vestoplast 888 (kindly provided by Evonik Resource Efficiency GmbH, Marl, Germany). Hereinafter, these materials will be referred to as VP708, VP750, VP792, and VP888, respectively. Their properties are shown in Table 1.

**Table 1:** Properties of the APAOs Used

name	molecular weight, *M*_w_ (g/mol)	viscosity at 190 °C (Pa s)
VP708	34,000	3 ± 1
VP750	92,000	50 ± 10
VP792	118,000	120 ± 30
VP888	104,000	120 ± 40

The PP matrix and the APAOs were mixed in an LTE 26-44 co-rotating twin-screw extruder (Labtech Engineering Co., Samutprakarn, Thailand). The revolution of the screw of the extruder was 90 1/min, and the revolution of the screw of the volumetric dosing unit was 12 1/min. The zone temperatures of the extruder were 160, 170, 175, 180, 180, 180, 185, and 190 °C from hopper to die, and the temperature of the die was 190 °C. The extruded filaments were granulated with an LZ-120/VS pelletizer (Labtech Engineering Co., Samutprakarn, Thailand). We prepared PP/APAO blends containing 5, 10, and 15 wt % of VP708, VP750, VP792, and VP888. The plain PP reference was also extruded to provide the same thermal history.

Sheet films were prepared from the blends with a 25–30C single-screw extruder and an LCR300 sheet-film line (Labtech Engineering Co., Samutprakarn, Thailand). For tensile drawing, 0.5 mm thick films were produced. In that case, the revolution of the screw was 90 1/min, and the take-up speed was 0.6 m/min. We produced 0.2 mm thick films for the drawing line, with a screw revolution of 120 1/min and a take-up speed of 1.8 m/min. In both cases, the zone temperatures of the extruder were 170, 180, 190, 195, and 200 °C from hopper to die, the die temperature was 200 °C, and the first roll of the sheet-film line was tempered to 85 °C. The revolution of the winder was set according to the diameter of the roll (between 6 and 11 1/min).

**Figure 1:**
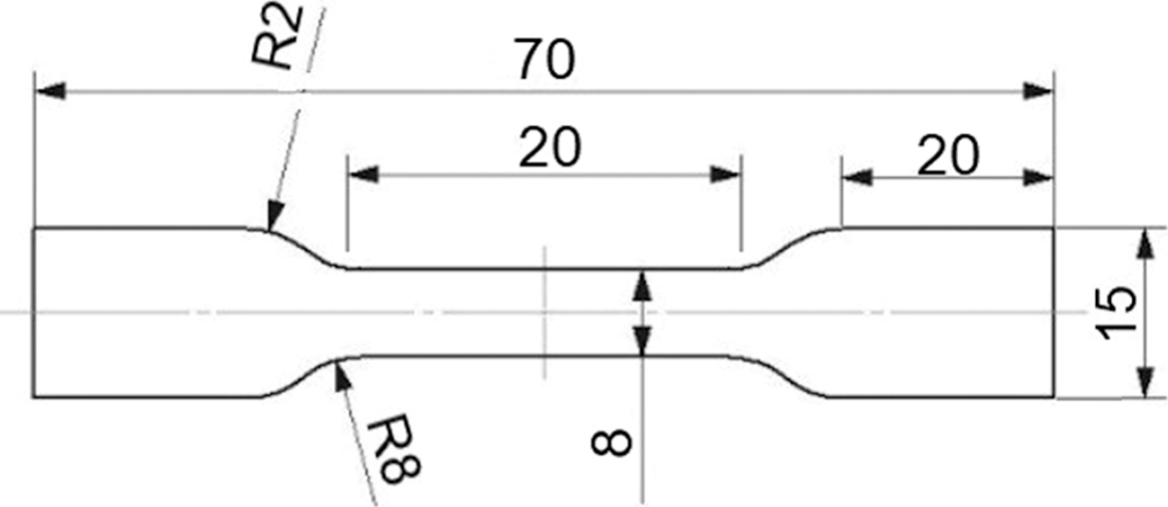
Specimens used in the drawing process.

Dumbbell specimens were punched out from the sheet films (Figure 1). The dimensions of the specimen were determined by the geometrical constraints of the heating chamber of the tensile testing machine; we were able to achieve a draw ratio of λ = 22. We designed the specimens with a measuring length of 20 mm, and with a width of 8 mm.

The specimens were drawn in the heating chamber of a Z250 tensile testing machine (Zwick GmbH, Ulm, Germany). The machine was equipped with wedge grips and a 20 kN load cell. The drawing temperature was 130 °C, and the drawing speed was 600 mm/min. We stored the specimens in the heating chamber for at least 10 min before drawing to set their temperature and left at least 2 min between closing the chamber door and the start of the drawing to maintain a constant temperature of 130 °C in the chamber. The specimens containing 10 wt % APAO were drawn to the draw ratio of λ = 5, 8, 9, 10, 12, 15, and 22, while the specimens containing 5 and 15 wt % APAO were drawn to the draw ratio of λ = 10. Force–strain curves during the drawing were recorded with the built-in software of the tensile testing machine.

The work of drawing was determined by calculating the area under the force–elongation curves recorded during the drawing process. We expressed the work of drawing in terms of the area of the cross-section of the specimens (the work of drawing was divided with the area of the specimens) to make the results comparable. Note that as the cross-section could not be continuously measured during the drawing process, the work of drawing was normalized to the initial cross-section of the specimens, similar to the method frequently used in the evaluation of tensile tests. This way, although the drawing curves do not represent the real stress in the specimens upon drawing, the curves became comparable to each other.

**Figure 2:**
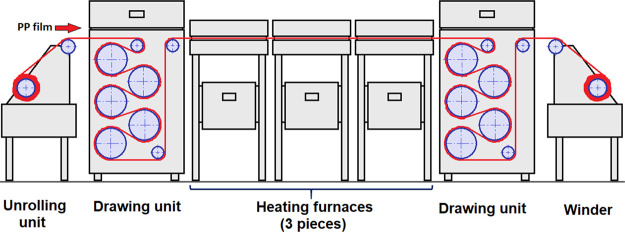
Schematic figure of the drawing line.

We also produced drawn tapes from the 0.2 mm thick films using a custom-made drawing line (Figure 2) to model the continuous operation of the industrial drawing process. We placed the rolls on an unrolling unit and pulled the film through three heating furnaces (together, their length was 1.8 m) with two drawing units. After cooling down, the drawn tapes were collected on a winder. The pulling speed of the first drawing unit was 2 m/min, and it was kept constant in all cases. The draw ratio was set by changing the take-up speed of the second drawing unit (as the draw ratio is equal to the ratio of the take-up speeds of the second and first drawing units). We produced drawn tapes with the draw ratios of λ = 8, 10, and 12, setting the take-up speed of the second drawing unit to 16, 20, and 24 m/min, respectively. The temperature of the tape was measured with a Testo 830-T2 thermometer (Testo SE&CO, Ledzkirch, Germany) right after it had left the last furnace. The temperature of the furnaces was set based on the results obtained from the thermometer to ensure that the tapes left the furnaces at 130 °C. The rolls of the second drawing unit were tempered to 20 °C.

Differential scanning calorimetry (DSC) was performed on the samples on a Q2000 DSC device (TA Instruments, New Castle, United States) with one heating ramp in the temperature range between 0 and 200 °C, with a heating rate of 10 °C/min in a 50 mL/min nitrogen atmosphere. The crystalline melting temperature was determined as the peak of the first heating curve, and crystalline melting enthalpy was determined as the area under the melting peak with respect to the baseline. The crystallinity of the samples was determined with the following equation:
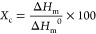
1where *X*_c_ is the crystallinity of the sample (%), Δ*H*_m_ is the melting enthalpy of the sample (J/g), and Δ*H*_m_^0^ is the melting enthalpy of a theoretically infinite PP crystallite (207 J/g^1^).

Tensile tests were conducted with a Z005 tensile testing machine (Zwick GmbH, Ulm, Germany). In the case of the drawn tapes, we cut out a 100 mm long sample from the middle of the tape, and the tensile tests were performed on these specimens with a measuring length of 40 mm. For undrawn samples, we used the specimens specified in Figure 1. In the case of the tapes drawn on the drawing line, we used 10x100 mm rectangular specimens. The test was performed with the drawing speed of 20 mm/min at room temperature in all cases. As the drawn width of the drawn samples could not be measured with conventional methods—as their width decreased greatly during the drawing process—we measured their width using a VHX-5000 light microscope (Keyence Ltd., Osaka, Japan). At least five specimens were tested in each case.

## 3 Results and Discussion

### 3.1 Tensile Drawing

**Figure 3:**
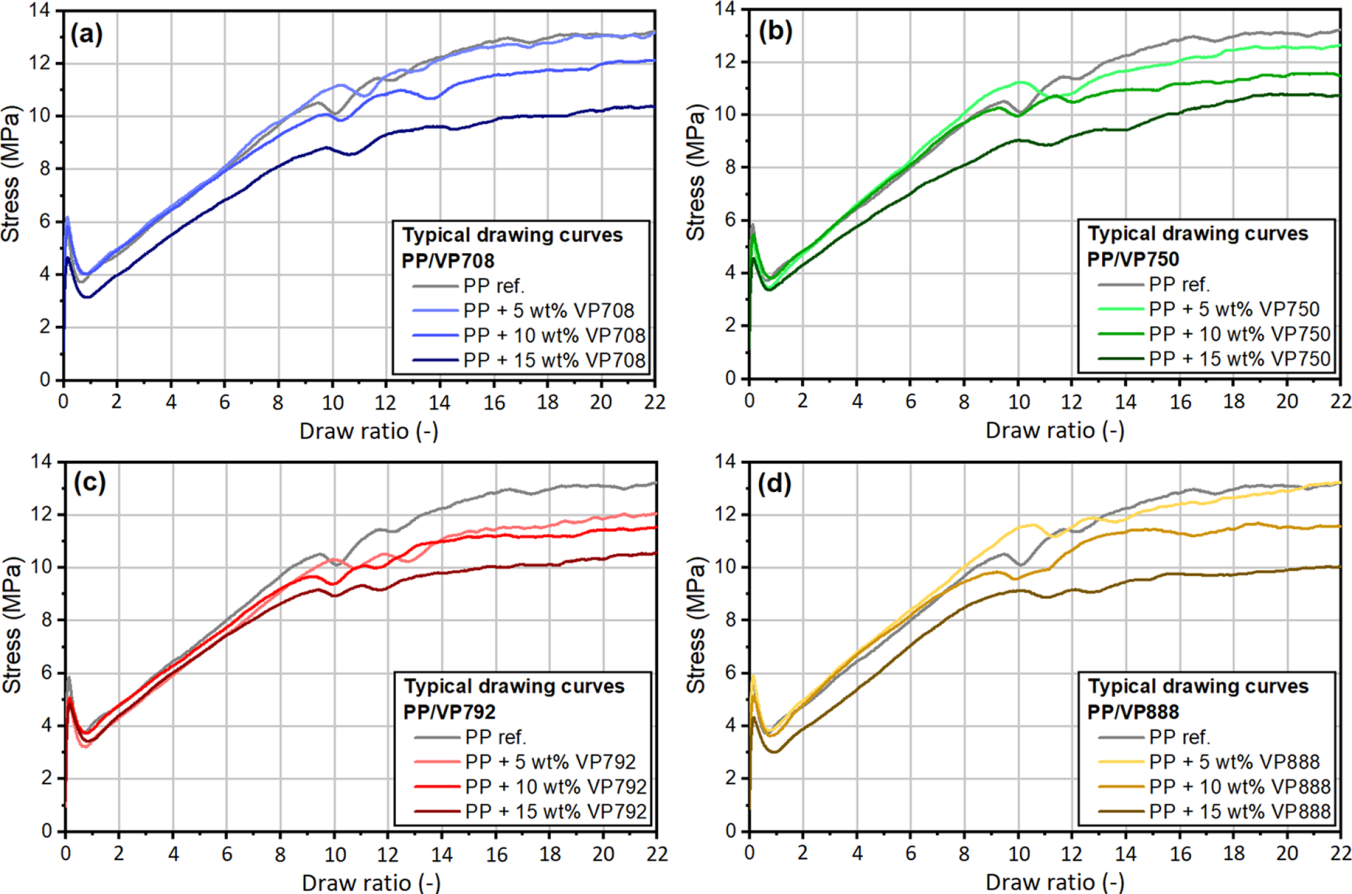
Typical drawing curves of PP/VP708 (a), PP/VP750 (b), PP/VP792 (c), and PP/VP888 (d) blends.

Typical drawing curves of the PP/APAO blends are presented in Figure 3. A change in the slope of the curves exists in the range of λ = 9...10. This probably indicates that the orientation of the crystalline phase completes around the draw ratio of λ = 9, above which only the orientation of the amorphous phase continues, as reported in the literature.^19,20^

Below the draw ratio of λ = 9, the curves of the blends with the APAO content of 5 and 10 wt % coincide with the curves of the pure PP, only the curves of the blends with 15 wt % APAO content differ from the others. Above λ = 9, however, a larger difference can be observed between the curves. This can be attributed to the fact that at λ = 9, the orientation of the crystalline phase completes, and above that, only the orientation of the amorphous phase continues. As APAOs—due to their mostly atactic nature—are almost completely amorphous, their molecules only can be present in the amorphous regions of the PP. Thus, in the region of the amorphous orientation, the effect of APAOs is more considerable.

**Figure 4:**
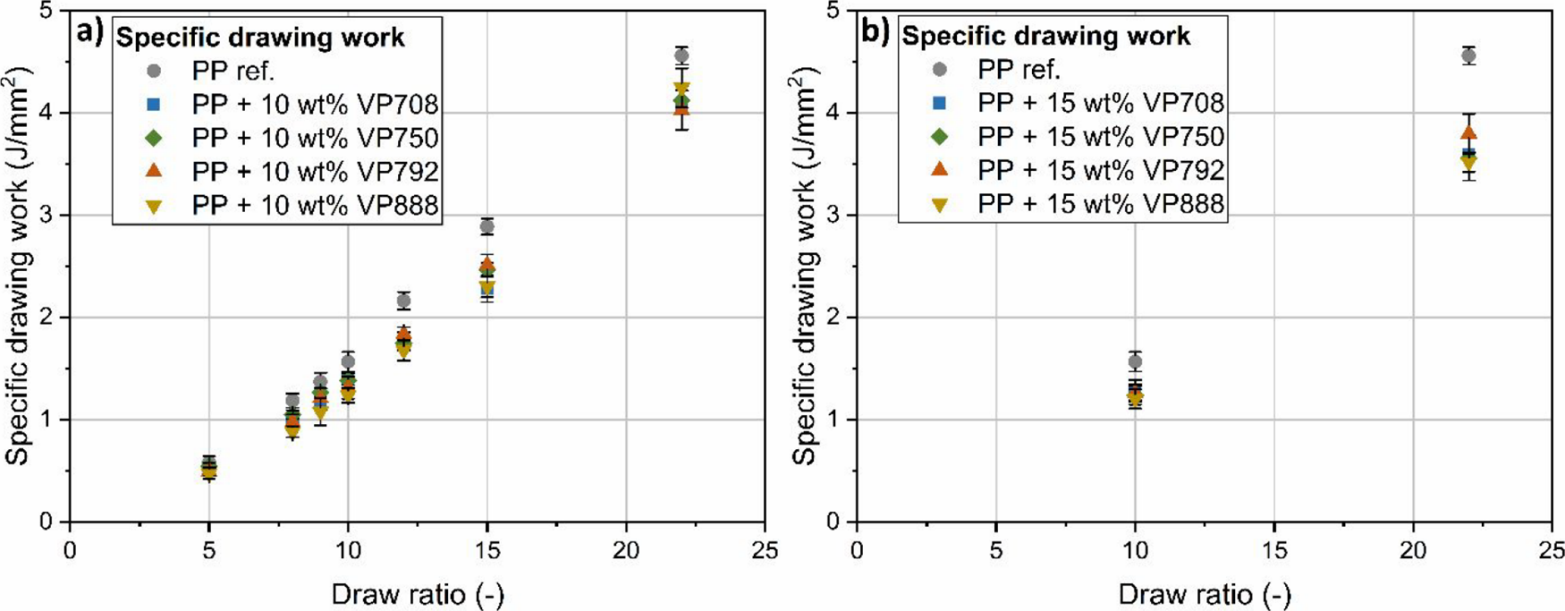
Specific work of drawing of the blends containing 10 (a) and 15 wt % APAO (b).

The relationship between the draw ratio and the specific work of drawing is linear (Figure 4). APAOs reduced the specific work of drawing as their shorter molecules facilitated the movement of the PP molecules during the drawing. No unambiguous difference was found between the effect of each APAO grade. Increasing the APAO content also caused a decrement in the specific work of drawing of the blends.

A small melting peak can be detected on the DSC curves of the drawn tapes between 150 and 154 °C. The size of this peak increased with increasing draw ratio. This peak can be attributed to the melting of the imperfect fibrils produced by the intense drawing. A few instabilities also can be seen on the DSC curves between 155 and 165 °C, especially in the case of tapes with higher draw ratios. This can be attributed to the increased relaxation phenomenon occurring in the highly drawn tapes at elevated temperatures. Relaxation decreases the molecular orientation of the tapes and reduces the length of the drawn tapes. This phenomenon could change the contact surface between the samples and the sample holder thus causing small fluctuations in the heat flow.

**Figure 5:**
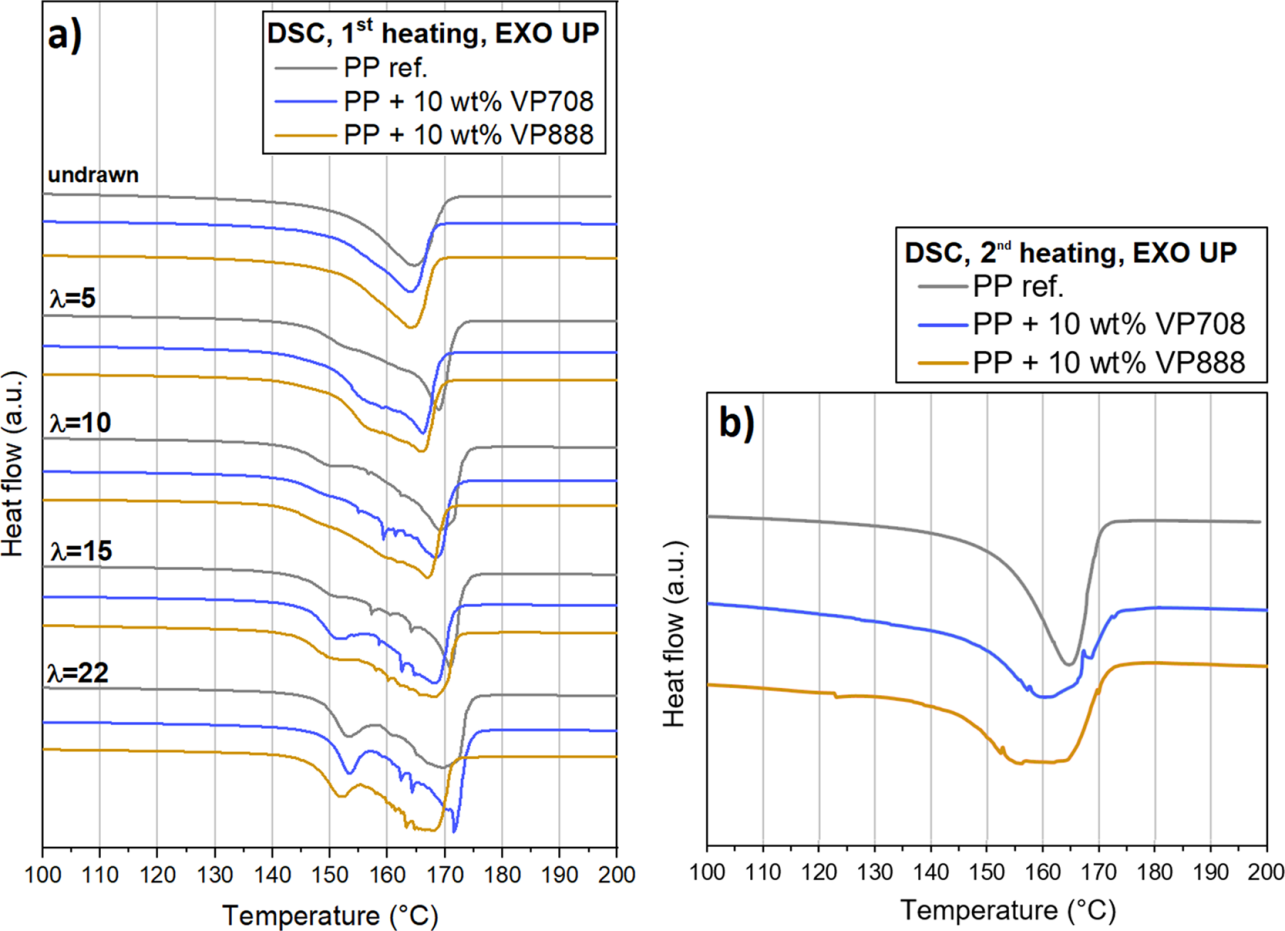
DSC curves from the first heating of the PP/VP708 and PP/VP888 blends with different draw ratios (a) and second heating curves of the blends (b).

**Figure 6:**
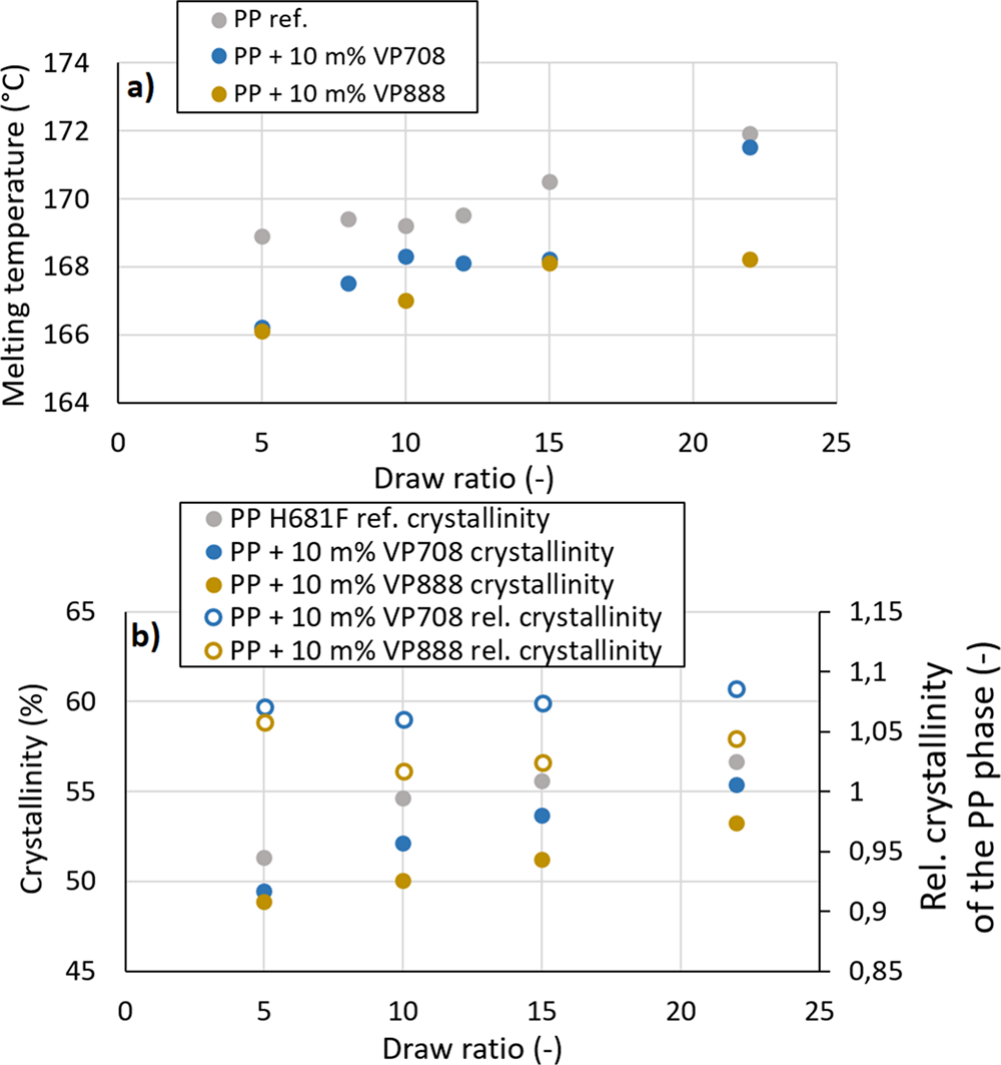
Crystalline melting temperatures (a) and crystallinity values (b) of the drawn PP/VP708 and PP/VP888 blends with different draw ratios.

Both crystalline melting temperature and enthalpy increased with increasing draw ratio. For the blends, however, both values were smaller compared to those of pure PP (Figure 5). This decrement was more significant for PP/VP708 blends than for PP/VP888 blends, even if pure VP888 is also capable of forming crystalline phases. It is possible that VP708—due to its lower molecular weight—was able to facilitate the movement of PP molecules more than the larger molecules of VP888, as VP708 with its smaller molecules acted as a diluent, and thus, increased the crystalline growth rate of PP, as reported by Chen et al.^24,25^ On the other hand, both VP708 and VP888 facilitated the crystal growth of PP, as the specific melting enthalpy (crystalline ratio divided by the PP content of the blend, taking the crystallinity of pure PP at the given draw ratio as 1) was more than 1 for each blend. This effect was also noticeable in the case of the undrawn samples, as relative crystallinity was 1.07 and 1.08 in the case of PP/VP708 and PP/VP888, respectively. The second heating cycle showed similar results (Figure 6b), as VP708 and VP888 decreased the overall crystallinity of the samples from 40.5 to 38.3 and 39.4%, respectively, while the relative crystallinity of the PP phase increased to 1.05 and 1.08 in the case of PP/VP708 and PP/VP888 blends, respectively.

**Figure 7:**
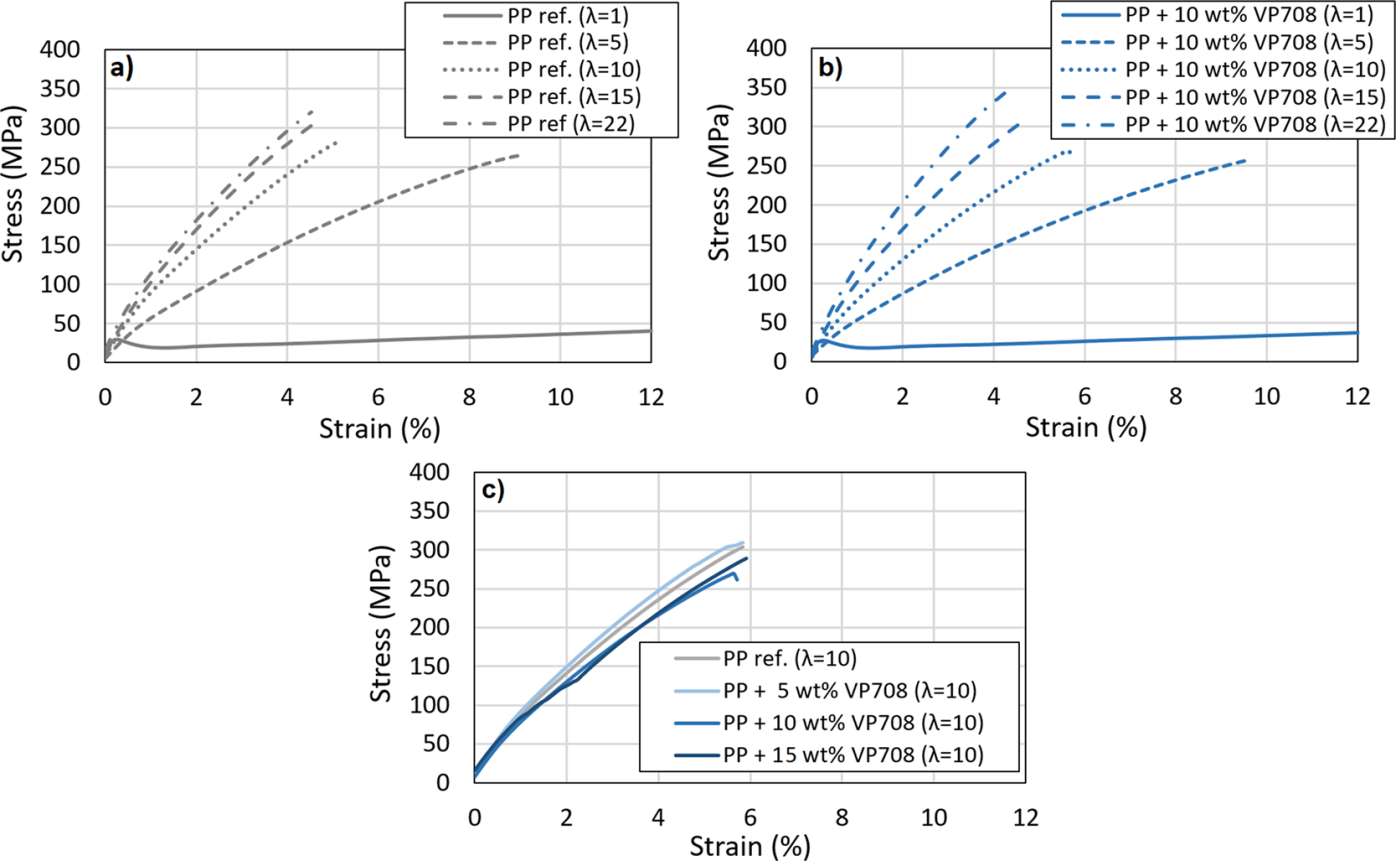
Typical tensile curves of plain PP (a), PP/VP708 blends with the additive content of 10 wt % at different draw ratios (b), and PP/VP708 blends with different APAO contents with the draw ratio of λ = 10 (C).

Drawn specimens failed abruptly during tensile tests (Figure 7). Both the draw ratio and APAO content changed the typical curves as expected. Although Figure 7 shows only the typical tensile curves of PP/VP708 blends, the curves of the other PP/APAO blends were similar.

**Figure 8:**
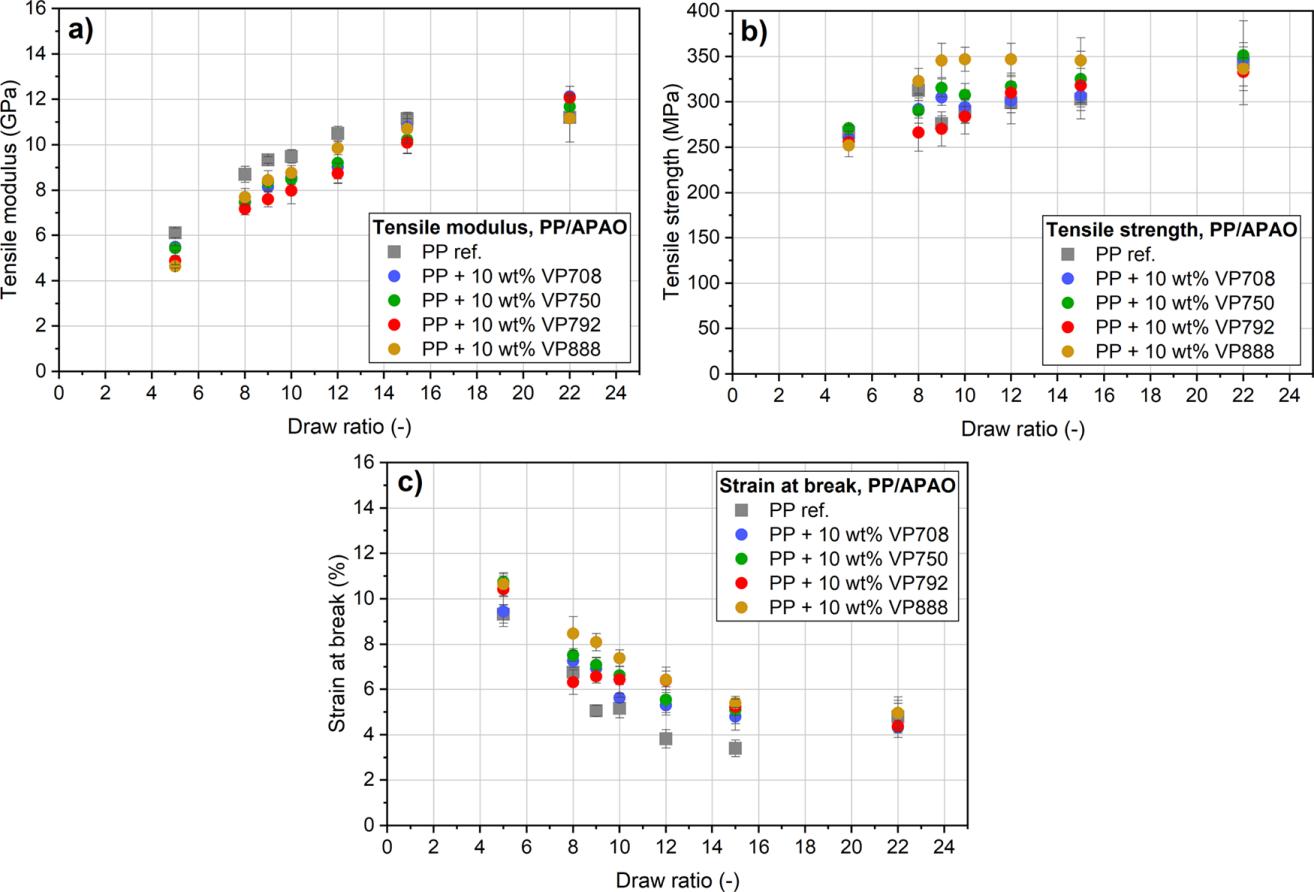
Tensile modulus (a), tensile strength (b), and strain-at-break (c) of the drawn PP/APAO blends with different draw ratios.

All APAOs decreased the tensile modulus similarly at all investigated draw ratios (Figure 8). This decrement, however, was less considerable at higher draw ratios (at λ = 15, but λ = 22 especially). Tensile modulus is attributed to the orientation of the amorphous phase,^19^ so it increased with increasing draw ratio, as amorphous orientation monotonously increases with increasing draw ratio. Tensile strength, however, increased only up to the draw ratio of λ = 9, as it is attributed to the crystalline orientation, and the orientation of the crystalline phase completes at the draw ratio of approximately λ = 9. VP888 increased the tensile strength, while this effect of the other APAOs could not be detected. A possible explanation is that the homopolymer regions of VP888 molecules were able to participate in the crystallization of the PP. Based on the results, there is no clear trend in the effect of the molecular weight of the APAOs on the tensile properties of the drawn tapes.

Increasing the APAO content reduced the tensile modulus of the tapes at the draw ratio of λ = 10 (Table 2). At λ = 22, however, 5 wt % of APAO increased the tensile modulus. Increasing the APAO content did not influence the tensile strength, except for VP888. The addition of APAOs increased the strain-at-break at the draw ratio of λ = 10, but no significant change can be seen at λ = 22.

**Table 2:** Results of the Tensile Tests

additive	additive content (wt %)	draw ratio (−)	tensile modulus (GPa)	tensile strength (MPa)	strain-at-break (%)
*PP ref.*		10	9.48 ± 0.29	289 ± 12	5.16 ± 0.41
*PP ref.*		22	11.2 ± 0.1	343 ± 46	4.79 ± 0.72
VP708	5	10	9.04 ± 0.39	311 ± 12	6.17 ± 0.37
VP708	5	22	11.8 ± 0.5	334 ± 24	2.92 ± 0.52
VP708	10	10	8.57 ± 0.32	294 ± 18	5.63 ± 0.20
VP708	10	22	12.1 ± 0.5	347 ± 8	4.33 ± 0.45
VP708	15	10	8.67 ± 0.49	311 ± 17	6.19 ± 0.41
VP708	15	22	11.7 ± 0.5	315 ± 11	3.84 ± 0.24
VP750	5	10	8.61 ± 0.55	306 ± 16	6.51 ± 0.32
VP750	5	22	12.0 ± 0.5	337 ± 26	5.69 ± 0.76
VP750	10	10	8.49 ± 0.24	308 ± 12	6.62 ± 0.42
VP750	10	22	11.7 ± 0.3	351 ± 14	4.96 ± 0.42
VP750	15	10	7.79 ± 0.43	280 ± 17	6.53 ± 0.24
VP750	15	22	11.2 ± 0.6	341 ± 9	4.83 ± 0.23
VP792	5	10	8.24 ± 0.28	296 ± 7	6.76 ± 0.43
VP792	5	22	12.2 ± 0.3	385 ± 13	5.87 ± 0.09
VP792	10	10	7.97 ± 0.57	284 ± 20	6.44 ± 0.79
VP792	10	22	12.1 ± 0.1	333 ± 16	4.38 ± 0.32
VP792	15	10	8.56 ± 0.49	294 ± 20	6.41 ± 0.44
VP792	15	22	11.7 ± 0.3	328 ± 22	4.68 ± 0.32
VP888	5	10	8.59 ± 0.20	338 ± 11	6.77 ± 0.28
VP888	5	22	12.2 ± 0.4	378 ± 33	5.23 ± 0.71
VP888	10	10	8.77 ± 0.31	347 ± 13	7.38 ± 0.37
VP888	10	22	11.2 ± 1.0	336 ± 24	4.96 ± 0.70
VP888	15	10	9.16 ± 0.53	316 ± 19	6.13 ± 0.27
VP888	15	22	11.5 ± 0.7	338 ± 14	4.83 ± 0.24

### 3.2 Continuous Drawing on a Stretching Line

**Figure 9:**
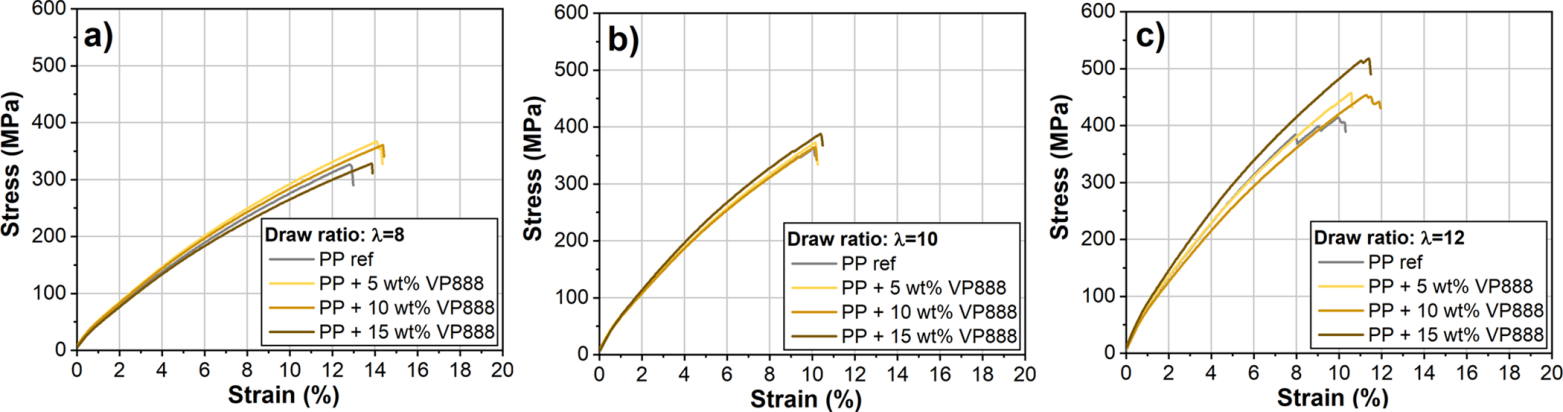
Typical tensile curves of the drawn tapes with different APAO content at the draw ratio of λ = 8 (a), λ = 10 (b), and λ = 12 (c).

The addition of VP888 simultaneously increased the tensile stress and strain-at-break of the drawn specimens, so we also examined the effect of VP888 on a continuous-operation stretching line. Typical tensile curves showed that the tensile strength was increased and strain-at-break was reduced after drawing, as expected (Figure 9). The curves did not show any sign of necking. At the draw ratios of 8 and 10, the specimens failed abruptly, while at the draw ratio of 12, some fibrillation was noticeable on the curves before the failure.

**Figure 10:**
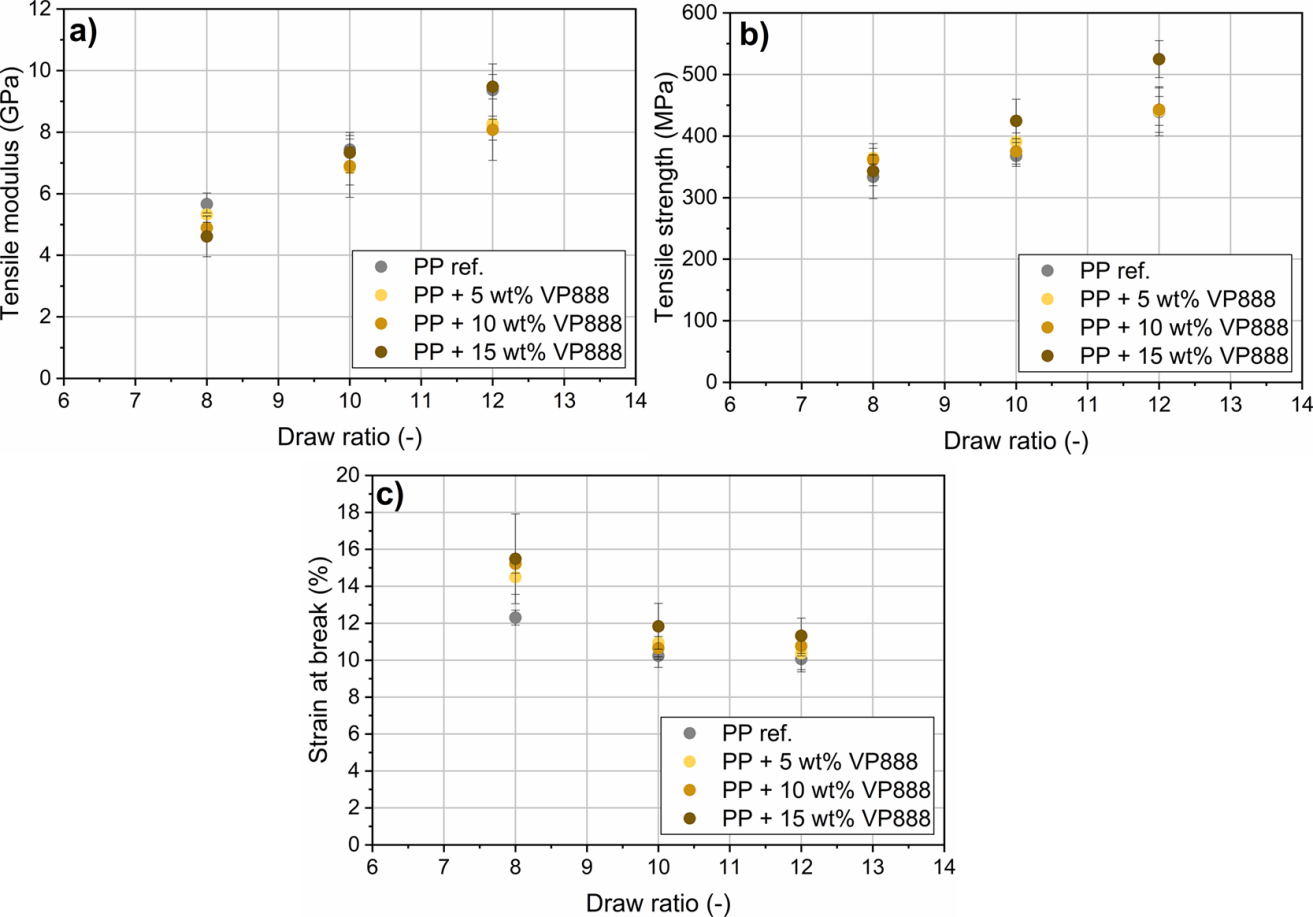
Tensile modulus (a), tensile strength (b), and strain-at-break (c) of the drawn PP/VP888 tapes at different draw ratios.

At the draw ratio of λ = 8, increasing VP888 content reduced the tensile modulus (Figure 10). At the draw ratios of λ = 10 and λ = 12, no clear tendency can be seen in the modulus values due to the higher deviations. It is important, however, that while the addition of VP888 did not reduce tensile strength (or, in some cases, even increased it), the strain-at-break increased with increasing VP888 content.

## 4 Summary

In this study, we investigated the possibilities of increasing the maximal strain of highly drawn PP tapes by blending PP with amorphous APAOs. We used four different APAOs in a wide range of molecular weights as additives and produced drawn PP tapes with different draw ratios and APAO content. Tapes were drawn in a heating chamber of a tensile testing machine and also on a custom-made drawing line.

APAOs reduced the crystalline ratio of the blends; the crystalline ratio relative to the PP content of the given blends, however, increased as the small APAO molecules acted like a plasticizer between the PP molecules thus increasing their ability to form highly ordered crystalline structures. All of the APAOs used decreased the work of drawing, and they increased the strain-at-break of the drawn tapes. Vestoplast 888 (the one with the higher molecular weight among the APAOs used and the only one with the crystalline phase), however, also increased the tensile strength between the draw ratios of λ = 8 and λ = 10. This effect of Vestoplast 888 was also experienced when the PP/APAO blend was drawn on a drawing line, which modeled the continuous operation of industrial-scale drawing.

Based on the results, APAOs seem promising additives to simultaneously improve the tensile strength and strain-at-break of drawn PP tapes. As those properties usually contradict each other, the PP/APAO blends can obtain great industrial importance, especially, as the reduced work of drawing can make the industrial-scale production of such tapes more economically feasible.
